# Photosynthesis in non‐foliar tissues: implications for yield

**DOI:** 10.1111/tpj.14633

**Published:** 2020-01-29

**Authors:** Andrew J. Simkin, Michele Faralli, Siva Ramamoorthy, Tracy Lawson

**Affiliations:** ^1^ Genetics, Genomics and Breeding NIAB EMR New Road, East Malling Kent ME19 6BJ UK; ^2^ School of Life Sciences University of Essex Wivenhoe Park Colchester CO4 3SQ UK; ^3^ School of Bio Sciences and Technology Vellore Institute of Technology Vellore 632014 India; ^4^ Present address: Department of Biodiversity and Molecular Ecology Research and Innovation Centre Fondazione Edmund Mach, via Mach 1 San Michele all'Adige (TN) 38010 Italy

**Keywords:** photosynthesis, Calvin–Benson cycle, sink capacity, wheat ears, non‐foliar organs, stomata

## Abstract

Photosynthesis is currently a focus for crop improvement. The majority of this work has taken place and been assessed in leaves, and limited consideration has been given to the contribution that other green tissues make to whole‐plant carbon assimilation. The major focus of this review is to evaluate the impact of non‐foliar photosynthesis on carbon‐use efficiency and total assimilation. Here we appraise and summarize past and current literature on the substantial contribution of different photosynthetically active organs and tissues to productivity in a variety of different plant types, with an emphasis on fruit and cereal crops. Previous studies provide evidence that non‐leaf photosynthesis could be an unexploited potential target for crop improvement. We also briefly examine the role of stomata in non‐foliar tissues, gas exchange, maintenance of optimal temperatures and thus photosynthesis. In the final section, we discuss possible opportunities to manipulate these processes and provide evidence that *Triticum aestivum* (wheat) plants genetically manipulated to increase leaf photosynthesis also displayed higher rates of ear assimilation, which translated to increased grain yield. By understanding these processes, we can start to provide insights into manipulating non‐foliar photosynthesis and stomatal behaviour to identify novel targets for exploitation in continuing breeding programmes.

## Significance Statement

Measurements of photosynthesis focus on leaves, however non‐foliar green tissue can make significant contributions to total plant carbon assimilation. This review evaluate photosynthesis and stomatal behavior in non‐foliar organs with a view to manipulate these processes for improved yield or quality.

## Introduction

**Figure 1:**
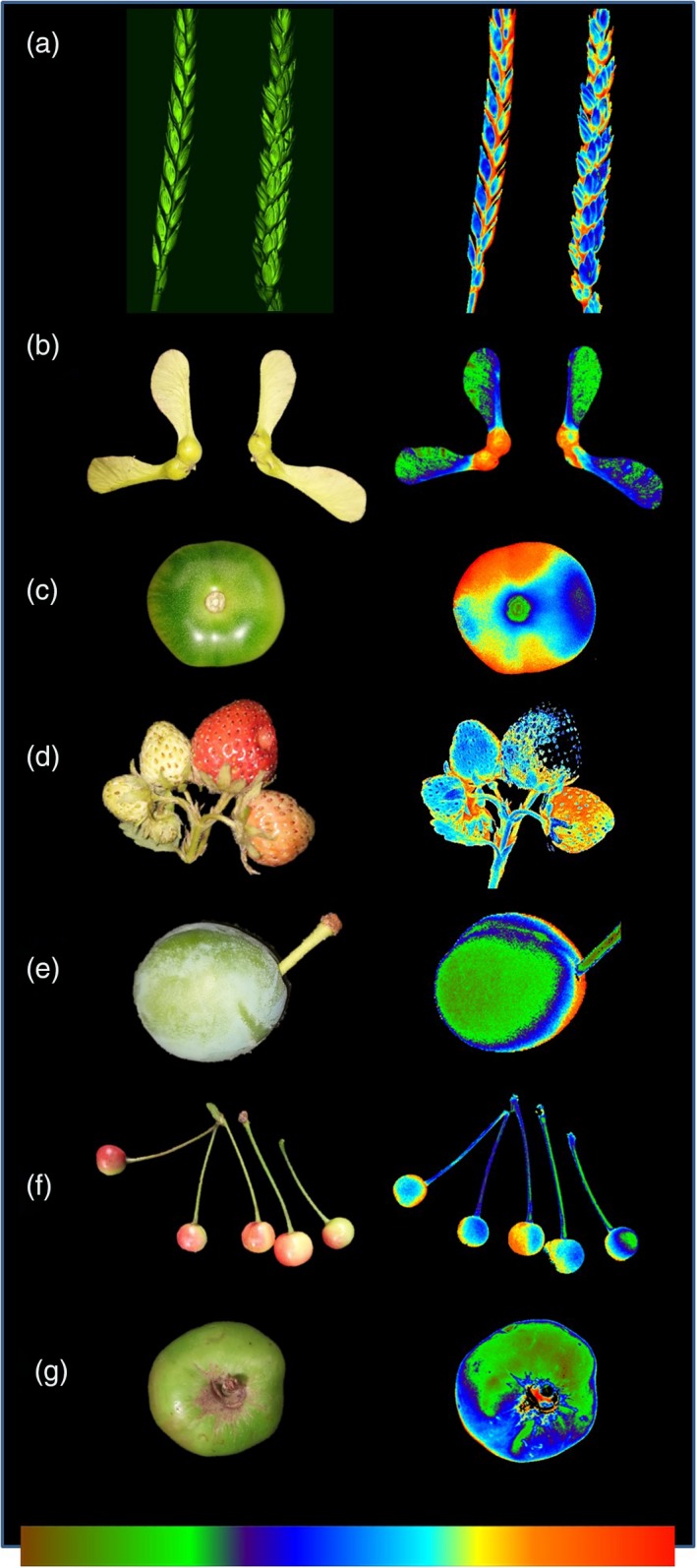
Chlorophyll fluorescence (CF) images of photosystem II (PSII) operating efficiency ($F_{q}^{\prime }/F_{m}^{\prime }$) in green non‐leaf tissue was used to demonstrate photosynthetic electron transport. CF images: (a) wheat ear; (b) sycamore seed pods; (c) tomato fruit; (d) strawberry fruit; (e) greengage; (f) cherries; and (g) apples. Colour scale bar represents an $F_{q}^{\prime }/F_{m}^{\prime }$ of: (a) 0.45–0.75; (b) 0.30–0.55; (c) 0.50–0.70; (d) 0.50–0.70; (e) 0.5–0.75; (f) 0.5–0.80; and (g) 0.45–0.70.

Photosynthesis in leaves is a well‐established and extremely well researched process whereby plants harvest the energy from sunlight and use this to convert CO_2_ into soluble carbohydrates, which are subsequently used for plant growth (Calvin and Benson, 1948; Bassham and Calvin, 1960; Raines, 2003; Biel and Fomina, 2015). Photosynthesis is responsible, therefore, either directly (through plant growth) or indirectly (through the food chain), for all food consumed worldwide. The majority of studies on photosynthesis often only consider photosynthesis in leaves, with little appreciation of potential carbon assimilation in other green non‐foliar tissue and its contribution to overall yield. With the predicted requirement to double food production by the year 2020 (WorldBank, 2008; RSOL, 2009; Tilman and Clark, 2015; FAO, 2017) and the fact that annual genetic gains in yield, using current breeding approaches, are reducing or slowing for many crops (Ray *et al.*, 2012; Ray *et al.*, 2013), research into photosynthesis and the processes associated with it are being increasingly recognized as potential novel targets for improving crop yield. Crop yield is determined by the cumulative rate of photosynthesis over the growing season. The maximum yield obtained (yield potential), defined as the yield obtainable when a crop is grown in optimal conditions with no biotic or abiotic stress (Evans and Fischer, 1999), is the result of three key determinants: (i) light capture; (ii) radiation use efficiency (RUE) or energy conversion efficiency (the product of which is biomass); and (iii) harvest index (HI, the partition of harvestable produce relative to plant biomass) (Reynolds *et al.*, 2009). Significant gains in both HI and light interception have been made over the last several decades, with considerable increases in HI following the green revolution and the introduction of dwarfing (*Rht*) genes (Gale and Youssefian, 1985; Calderini *et al.*, 1995). The current focus is on RUE (Reynolds *et al.*, 2009; Parry *et al.*, 2011), which is primarily photosynthesis and the conversion of light energy into fixed carbon. Several recent studies have demonstrated that improving diverse aspects of photosynthesis in leaf tissue, including altering key enzymes within the Calvin–Benson cycle (CBC) (Lefebvre *et al.*, 2005; Simkin *et al.*, 2015; Driever *et al.*, 2017; Simkin *et al.*, 2017a), electron transport (Chida *et al.*, 2007; Simkin *et al.*, 2017b; Yadav *et al.*, 2018; Ermakova *et al.*, 2019), photorespiration (Timm *et al.*, 2012; López‐Calcagno *et al.*, 2018) and the kinetics of non‐photochemical quenching (NPQ) (Kromdijk *et al.*, 2016; Glowacka *et al.*, 2018) can improve yield potential in both glasshouse‐ and field‐grown plants (Simkin, 2019; Simkin *et al.*, 2019). Leaves are not the only location within the plant where photosynthesis occurs, however, with evidence that petioles and stems (Hibberd and Quick, 2002), seeds (Schwender *et al.*, 2004), fruit (Hetherington *et al.*, 1998; Carrara *et al.*, 2001; Hiratsuka *et al.*, 2015; Sui *et al.*, 2017), *Triticum aestivum* (wheat) ears (Maydup *et al.*, 2010), and the husks of *Zea mays* (corn) (Pengelly *et al.*, 2011) all photosynthesize and may provide significant and alternative sources of the photoassimlates, essential for optimal yield. Figure 1 illustrates chlorophyll fluorescence imaging of the operating efficiency of photosystem II (PSII) photochemistry ($F_{q}^{\prime }/F_{m}^{\prime }$) in non‐leaf tissues, which is indicative of functional electron transport in these green non‐leaf organs. To date little data exist on how potential manipulation of photosynthetic processes may impact these chlorophyll‐containing tissues.

The majority of studies that have examined photosynthesis in non‐foliar tissue have assumed and described a photosynthetic pathway similar to that of the mesophyll. One key difference in non‐foliar tissue photosynthesis is the fact that there are two potential major sources of CO_2_. First, ribulose‐1,5‐bisphosphate carboxylase (Rubisco) assimilates atmospheric CO_2_ that diffuses into the cells through the stomatal pores, leading to the production of sugars via the CBC, similar to the CO_2_ pathway in leaf (C3) tissue. Second, CO_2_ released by mitochondrial respiration can be the main supply of CO_2_ and is refixed (recycling photosynthesis; Aschan and Pfanz, 2003; Millar *et al.*, 2011), and there is limited diffusion and supply of external CO_2_. Although stomata are present in various numbers on some non‐foliar tissues their function has not been fully evaluated, and the amount of photosynthesis that relies on the atmospheric supply of CO_2_ through these pores is not currently known. In this review we focus on photosynthesis in non‐foliar tissues and the potential contribution to yield, as well as the role of stomata in this process. Before discussing the possibility to manipulate non‐foliar photosynthesis for improved productivity or nutritional quality, we first provide an overview of what is known about photosynthesis in various organs, focusing on stems and fruits as well as various parts of cereals.

## Photosynthesis in stems

Stems act as temporary storage sites for photoassimilates from leaves and carry out photosynthesis in their own right (Aschan and Pfanz, 2003). In *Solanum lycopersicum* (tomato), chlorophyll levels were found to be higher in the upper parts of the stem than in the lower parts of the stem (Xu *et al.*, 1997), and a comparison of the photosynthetic activity of various plant parts found the entire stem accounted for up to 4% of photosynthetic activity (Hetherington *et al.*, 1998). The contribution of stem photosynthesis to yield has been demonstrated in *Gossypium hirsutum* (cotton) by Hu *et al. *(2012) who reported that keeping the main stem in darkness reduced seed weight by 16% (Hu *et al.*, 2012). These findings were supported by Simbo *et al. *(2013), who showed that when light was excluded from the stem of defoliated *Adansonia digitata* L. (African baobab) and *Ricinus communis* (castor bean) a reduction in bud dry weight was observed, providing further evidence for the importance of the stem for providing photoassimilates for plant development and growth. In some plants, such as *Justicia californica,* flowers and fruits develop in the absence of leaves, where the stem is the only photosynthetically active tissue (Tinoco‐Ojanguren, 2008; Ávila *et al.*, 2014), also highlighting the role of stem photosynthesis for reproductive success. This is emphasized further by a reported stem photosynthesis equivalent to 130% of leaf levels in this species (Tinoco‐Ojanguren, 2008), whereas in other species, rates of between 16 and 60% relative to leaf levels have been reported (Ehleringer *et al.*, 1987; Ávila *et al.*, 2014). In the woody plant *Eucalyptus* photosynthesis in chlorophyll‐containing tissue, chlorenchyma, located beneath the periderm layer (Pfanz *et al.*, 2002; Manetas, 2004), known as corticular photosynthesis (CP), contributed 11% of total photosynthate to plant growth, demonstrating the contribution of CP to eucalyptus growth (Cernusak and Hutley, 2011).

Stem photosynthesis is particularly important in deciduous species. In the summer‐deciduous, green‐stemmed Mediterranean shrub *Calicotome villosa*, the total branch photosynthesis is higher in the summer because of an absence of leaves, and green‐stem photosynthesis outcompetes leaf photosynthesis on an annual basis (Yiotis *et al.*, 2008). In the desert ephemeral *Erigonum inflatum* substantial photosynthesis was demonstrated in the inflated stems, despite the fact that these contained only half the chlorophyll and nitrogen content of the leaves (Osmond *et al.*, 1987). Internal CO_2_ concentrations in these stems was reported to be extremely high. Interestingly, fixation of this internal CO_2_ was between six and 10 times less than the fixation of atmospheric CO_2_; however, although small, this additional internal CO_2_ pool facilitated high water‐use efficiency (WUE, measured as water lost relative to carbon gained) as a result of no water loss through stomata for this carbon gain. Greater WUE was further enhanced in this species by smaller stem stomata that are more responsive to temperature and high vapour pressure deficit (VPD), compared with their leaf counterparts (Osmond *et al.*, 1987). The importance of stem photosynthesis in desert species is supported by a more recent study by Avila‐Lovera *et al. *(2017), who examined 11 green‐stemmed desert plants and revealed coordination between stem photosynthesis and hydraulics similar to that observed in leaves, with an even tighter relationship during the dry season, facilitating additional carbon gain and potential mechanisms for enhanced drought tolerance. Furthermore, stem photosynthetic rates were higher during the dry season when leaves were lost and light interception by the stems was increased, due to the absence of foliage (Avila‐Lovera *et al.*, 2017). Together these studies illustrate the importance and annual contribution of stem photosynthesis to overall carbon gain, which not only contributes to the survival of plants growing in dry and hot environments, but also supports the notion that stem photosynthesis may contribute significantly to yield, and that this contribution may be more important under conditions such as reduced water availability, high temperatures and high VPD. To date, however, there have been limited studies that have evaluated the importance of stem photosynthesis to yield in key crop species. Therefore, although stem photosynthesis may represent a potential novel target to support enhanced photosynthetic carbon gain, particular under conditions of water stress (such as those predicted under climate change for certain agricultural areas), more quantitative information on stem performance in crops is needed to evaluate and fully exploit this process.

## Fruit photosynthesis

Fruit photosynthesis is particularly interesting, as many species (e.g. tomato) undergo a shift from green photosynthetic (or partial photosynthetic) to fully heterotrophic metabolism on ripening (Lytovchenko *et al.*, 2011). As early as 1974, Tanaka and coworkers conducted shading experiments on tomato fruits and showed that fruit photosynthesis contributes to net sugar accumulation and growth (Tanaka *et al.,* 1974), and from this work concluded that photosynthesis contributed between 10 and 15% of the total fixed carbon, which was later confirmed by Hetherington *et al. *(1998) and Obiadalla‐Ali *et al. *(2004). In addition to showing a similar photosynthetic function to leaves, developing tomato fruit have also been reported to have approximately 41% of the photosynthetic electron transport capacity of leaf tissue (Piechulla *et al.*, 1987). Recent proteomic analysis has demonstrated that all of the components of the CBC and photorespiratory cycle accumulate at the protein level in tomato fruit (Barsan *et al.*, 2010; Barsan *et al.*, 2012). The major light‐harvesting proteins, including the thylakoid membrane light‐harvesting complex proteins of PSI (*psaA*) and PSII (*psbA*), and the chlorophyll *a*/*b* binding proteins, have also been observed (Piechulla *et al.*, 1986; Lemaire‐Chamley *et al.*, 2005), in conjunction with plastocyanin, cytochrome *f*, cytochrome *b*, ferredoxins, Rieske iron sulphur protein (Piechulla *et al.*, 1987; Livne and Gepstein, 1988; Cheung *et al.*, 1993; Aoki *et al.*, 1998) and the CBC proteins, Rubisco and fructose 1,6‐bisphophate aldolase (FBPaldolase) (Barsan *et al.*, 2010; Steinhauser *et al.*, 2010). Rubisco assays have also demonstrated that the enzyme is active in tomato fruit (Willmer and Johnston, 1976; Bravdo *et al.*, 1977; Laval‐Martin *et al.*, 1977; Piechulla *et al.*, 1987; Sugita and Gruissem, 1987).

Despite the fact that transcriptomic and metabolomic analyses have revealed high expression levels of many of these photosynthetic genes in tomato fruit, and have shown that photosynthetic carbon assimilation in these organs makes an important contribution to early fruit development (Wang *et al.*, 2009), many studies do not agree that these fruit are net assimilators of CO_2_ (see Blanke and Lenz, 1989; Carrara *et al.*, 2001). Lytovchenko *et al. *(2011) used antisense technology to reduce expression of the chlorophyll biosynthesis gene glutamate 1‐semialdehyde aminotransferase, which resulted in a reduced photosynthetic rate; however, fruit size and metabolite levels remained unchanged. These authors suggested that transport of photosynthate from leaves compensated for any reduction in fruit localized photosynthetic rates and proposed that fruit photosynthesis is dispensable. However, a delay in seed development was observed, suggesting that localized CO_2_ fixation/re‐assimilation may be important for seed formation (Lytovchenko *et al.*, 2011). In contrast, another study demonstrated that decreased expression of fruit chloroplastic fructose‐1,6‐bisphosphatase (FBPase) resulted in a 15–20% negative impact on fruit development (Obiadalla‐Ali *et al.*, 2004). Lytovchenko *et al. *(2011) suggested that these contradictory results could be explained by different promotor specificity and/or the impact of reduced FBPase activity later in the development of the fruit.

**Figure 2:**
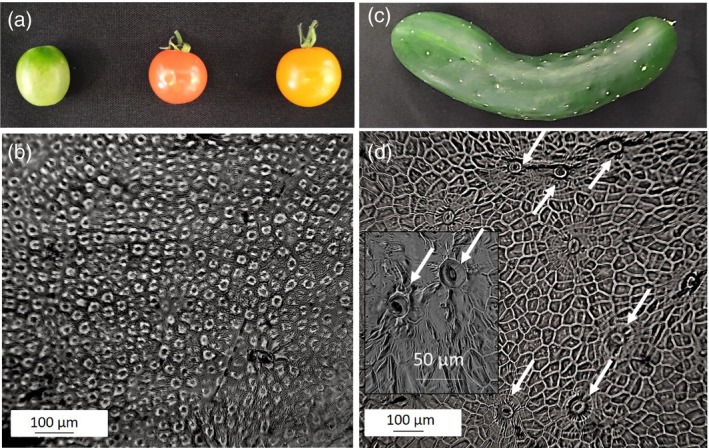
Example of epidermal impressions taken from tomato (b) and cucumber (d). Photographs of the fruit are presented in (a) and (c). Stomata were absent from the epidermis of tomato (b), whereas a relatively high stomatal density is illustrated in cucumber (d, with the inset showing a magnified stomatal complex).

Although it is evident that photosynthesis occurs in fruits, the extent and importance is not clear. The fact that tomato fruit lack stomata (Vogg *et al.*, 2004) (Figure 2) implies that photosynthesis in these organs relies exclusively on CO_2_ liberated from mitochondria, that no ‘new’ carbon is fixed and that photosynthesis functions to re‐assimilate CO_2_ (recycling photosynthesis) that would otherwise be lost. This is supported by the reported accumulation of transcripts in tomato loculare tissue associated with photosynthesis, clearly demonstrating photosynthetic capacity, but alongside high measured respiration rates (Lemaire‐Chamley *et al.*, 2005). CO_2_ generated by the oxidative pentose pathway is re‐assimilated by the CBC in a manner previously reported in green seeds of *Brassica napus* (oilseed rape) (Schwender *et al.*, 2004). It has been reported that these photosynthesis‐specific transcripts are regulated by transcription factors in a similar way to those in leaf tissue (Hetherington *et al.*, 1998; Carrara *et al.*, 2001); however, a number of authors have reported the existence of some fruit‐specific regulation of photosynthetic genes (Piechulla *et al.*, 1987; Piechulla and Gruissem, 1987; Sugita and Gruissem, 1987; Manzara *et al.*, 1993), and Cocaliadis *et al. *(2014) suggested that this is likely to optimize photosynthetic function for fruit development. This specificity therefore provides a potential route for manipulating key photosynthetic genes specifically in fruit to enhance development, yield or nutritional quality.

In summary, it appears that photosynthetic carbon assimilation does take place in green immature tomato fruit and that this relies almost exclusively on respired CO_2_, and that any reductions in the rate of photosynthesis in these organs can be compensated for by the upregulation of leaf photosynthesis (Nunes‐Nesi *et al.*, 2005; Araújo *et al.*, 2011) and increased imported photoassimilates from leaves. Such import cannot compensate for the losses of fruit photosynthesis for seed set, establishment and development (Lytovchenko *et al.*, 2011). Therefore, altering fruit photosynthesis could provide advantages of early seed set, as well as maintaining yield, particularly under conditions of stress when leaf photosynthesis may be compromised.

Tomato photosynthesis is restricted to the green phases of development up until chloroplast‐to‐chloroplast differentiation, which is marked by the loss of chlorophyll, the degradation of the thylakoid membranes, and a strong decrease in the levels of photosynthesis‐associated transcripts and proteins (Harris and Spurr, 1969a; Harris and Spurr, 1969b; Cheung *et al.*, 1993; Barsan *et al.*, 2012), after which the fruit continues to develop and ripen. This is similar for other fruits such as *Capsicum annum* (pepper) (Steer and Pearson, 1976), *Citrus unshiu* (satsuma mandarin) (Hiratsuka *et al.*, 2015), blueberry (Birkhold *et al.*, 1992); coffee (*Coffea arabica*) (Cannell, 1985; Lopez *et al.*, 2000); *Prunus tomentova* (plum) (Aoyagi and Bassham, 1984); the ornamental plant *Arum italicum* (Ferroni *et al.*, 2013) and *Jatropha curcas* (Ranjan *et al.*, 2012). In satsuma mandarin, it has been demonstrated that photosynthesis occurs in these fruits, is greater at low irradiances, and increases with increasing [CO_2_] supplied through fully developed stomata in the rind of satsuma (Hiratsuka *et al.*, 2015). The fact that stomata can be found in densities of about 72 mm^−2^ in immature *Jatropha curcas* fruit suggests that new carbon can be assimilated through these tissues (Ranjan *et al.*, 2012). In this case, given the importance of fruit photosynthesis in the absence of leaves, increasing the stomatal density could increase CO_2_ uptake and boost photosynthetic rates in fruit, with a positive impact on yield.

*Cucumis sativus* (cucumber) is fundamentally different to tomato and other coloured fruit, remaining green through to full maturity, with a surface area equivalent to a fully expanded leaf (Sui *et al.*, 2017). An analysis of gene expression found a number of CBC enzymes (SBPase, FBPase, rbcL, rbcS) and light‐harvesting complex proteins of PSI (*Lhca*) and PSII (*Lhcb*) expressed in the exocarp (Sui *et al.*, 2017). Interestingly, unlike tomato, stomata are found on the epidermis of cucumbers (Figure 2), although Sui *et al. *(2017) reported a layer of epicuticular waxes around the guard cells that may reduce function. However, the presence of these pores on the fruit surface suggests, in the case of cucumber at least, that these fruits are capable of assimilating some CO_2_ directly from the atmosphere. Their physiology also suggests that photosynthesis can occur from the re‐assimilation of respiratory CO_2_. Cucumber fruits have been shown to have both high photosynthetic and respiratory rates (Todd *et al.*, 1961), and a recent study demonstrated that fruit photosynthesis contributed 9.4% of its own carbon requirements whereas 88% of respiratory CO_2_ in fruit was refixed (Sui *et al.*, 2017). Improving photosynthetic efficiency in fruit, therefore, has the potential to increase the fruit carbon contribution for growth through both recycling respiratory CO_2_ and atmospheric assimilation, which could in turn directly impact WUE. The need to maintain or increase fruit yield (or fruit size) whilst using less water cannot be underestimated given current environmental changes.

### Are stomata important in fruit photosynthesis?

It is important to note that although stomata are routinely found on the surface of some fruit and are of a similar size to stomata found on respective leaves, the numbers are generally significantly lower compared with those found in leaf tissue (Blanke, 1998). For example, Blanke and Lenz (1998) reported that the number of stomata on mature *Malus domestica* (apple) fruit was 30 times less abundant than the stomata found on apple leaves. Stomatal numbers are fixed at anthesis and as the fruit expands during growth, they become more dispersed (Hieke *et al.*, 2002; Hetherington and Woodward, 2003). Although it has been reported that stomatal density in fruit typically represents 1–10% of the frequency found in corresponding leaf tissue (Sánchez *et al.*, 2013), these numbers can vary greatly depending on the species. In *Persea americana* (avocado), the number of stomata on the fruit represent 14% of the number on the leaf (Blanke, 1992), whilst in green coffee fruit this number is 13–23% (Cannell, 1985), whereas in oranges the stomatal densities can reach up to 30% of those found on leaves (Moreshet and Green, 1980). To date, most studies have focused on the presence of stomata on various fruit tissue but have not fully demonstrated the functionality. If functional, however, the presence and stomatal densities reported above suggest that under certain conditions, in certain plants at least, stomata may play a role in gas exchange and therefore manipulating stomatal numbers through developmental mechanisms or transgenic approaches has the potential to change CO_2_ assimilation rates and yields. In other plants, however, the contribution of stomata to assimilation appears to be negligible compared with recycling photosynthesis. In these plants, we cannot rule out that the role of stomata is primarily for evaporative cooling. Although not directly related to CO_2_ uptake, this process may help maintain fruit temperature at an optimal level for recycling photosynthesis, thereby maximising CO_2_ recovery.

## Seed and embryo photosynthesis

The fruit pericarp is not the only non‐foliar green tissue that is capable of photosynthesis. The embryos of many taxa contain significant quantities of chlorophyll, which persists until maturity (Yakovlev and Zhukova, 1980; Simkin *et al.*, 2010; Puthur *et al.*, 2013; Smolikova and Medvedev, 2016). This group includes model species (*Arabidopsis thaliana*) and important crops such as *Cicer arietinum* L. (chickpeas), coffee, cotton, *Glycine max* L. (soybean), oilseed rape, *Pisum sativum* L. (peas), and *Vicia faba *L. (broad beans). These embryos, first referred to as chloroembryos by Palanisamy and Vivekanandan (1986), contain all the photosynthetic complexes of PSI and PSII, cytochrome *b*_6_ *f* complex and ATP synthase (Weber *et al.*, 2005; Allorent *et al.*, 2015; Kohzuma *et al.*, 2017). Chloroembryos have been shown to photosynthesize (Smolikova and Medvedev, 2016; Smolikova *et al.*, 2017), and confirmation of carbon fixation is supported by the activity of the CBC enzymes NADP‐glyceraldehyde‐3‐phosphate dehydrogenase (GAPDH) in the chloroembryo chloroplasts of oilseed rape and pea (Smith *et al.*, 1990; Eastmond *et al.*, 1996) and fructose‐1,6‐bisphosphatase (FBPase) in oilseed rape (Kang and Rawsthorne, 1996). Furthermore, Rubisco has also been shown to be active in the seeds of soybean (Allen *et al.*, 2009), oilseed rape (Hills, 2004; Ruuska *et al.*, 2004), broad bean (Willmer and Johnston, 1976) and *Trigonella foenum‐graecum* (Willmer and Johnston, 1976).

The contribution of photosynthesis in embryos may be different to that described above for fruit, as it has been reported that embryo photosynthesis contributes a significant amount of oxygen, which fuels energy‐generating biochemical pathways, including respiration and glycolysis (Ruuska *et al.*, 2004; Borisjuk *et al.*, 2005; Tschiersch *et al.*, 2011; Galili *et al.*, 2014). The role of photosynthesis in chloroembryos has also been associated with the rapid synthesis of ATP and NADPH for the synthesis of complex carbohydrates, fatty acids and proteins (Asokanthan *et al.*, 1997; Wu *et al.*, 2014). It has been reported that a key source of carbon is sucrose, imported from the leaves (Asokanthan *et al.*, 1997), which is respired by the seed, releasing CO_2_ (Ruuska *et al.*, 2004; Smolikova and Medvedev, 2016) within chloroembryos, which is subsequently efficiently re‐assimilated and thus directly affects the carbon economy of the seed (Puthur *et al.*, 2013).

In oilseed rape, seed photosynthesis plays a role in the accumulation of storage lipids (Eastmond *et al.*, 1996; Ruuska *et al.*, 2004). Interestingly, Rubisco acts in a distinctive context, without the CBC, to increase the carbon‐use efficiency for the synthesis of oil (Schwender *et al.*, 2004). This unique pathway generates 20% more acetyl‐CoA than glycolysis, reducing the loss of CO_2_ and increasing the availability of acetyl‐CoA for fatty acid biosynthesis (Schwender *et al.*, 2004). In the embryos of legumes, including pea, the main CO_2_‐refixing enzyme is phosphoenol pyruvate (PEP) carboxylase (Golombek *et al.*, 1999) suggesting that CO_2_ is refixed at the site of origin. In the case of pea, a small spherical seed with a green embryo within a seed pod, only a fraction of light reaches the photosynthetically active tissue. The light is attenuated by the pod, reflecting or absorbing as much as 75% of the sunlight. Only 32% of the remaining sunlight (approximately 8% of photosynthetic active radiation (PAR)), penetrates the pod and seed coat to reach the surface of the embryo; however, this is enough to drive photosynthesis with the highest electron transport rates reported in the seed coat (Tschiersch *et al.*, 2011). In addition to seed photosynthesis, pea pods also photosynthesize in two distinct layers. First, the outer layer, comprising chlorenchyma and mesocarp, assimilates CO_2_ from the atmosphere and second, the inner epidermis lining of the pod cavity reassimilates the CO_2_ released by the embryonic respiration into the pod cavity (Atkins *et al.*, 1977). Rubisco activity has also been detected in the pod wall of pea embryos, although this activity is 10–100 times lower than that detected in the leaf tissue (Hedley *et al.*, 1975).

## Importance of photosynthesis in non‐foliar cereal organs

In cereals, although leaf photosynthesis plays a central role in biomass accumulation and yield formation over the entire growing season (Fischer *et al.*, 1998; Gu *et al.*, 2014), the photosynthetic activity of the ear has been shown to dramatically contribute to the pool of carbohydrates translocated to the developing grains over the post‐anthesis stages (Tambussi *et al.*, 2005; Tambussi *et al.*, 2007; Maydup *et al.*, 2010; Sanchez‐Bragado *et al.*, 2014). Although on an area basis, the ear CO_2_ assimilation rate is lower than that of the flag leaf (Tambussi *et al.*, 2005; Tambussi *et al.*, 2007; Zhou *et al.*, 2016), experimental evidence suggests that in bread and durum wheat, ear photosynthesis can contribute to the individual grain weight yield component by up to 70% in a large range of genotypes (Maydup *et al.*, 2010) and contrasting environments (Sanchez‐Bragado *et al.*, 2014). Similarly to wheat, in *Hordeum vulgare* (barley), shading experiments revealed a significant contribution of the ear (up to 50%) to grain weight and therefore yield (Bort *et al.*, 1994). In the next few sections we focus on different aspects of ear photosynthesis and the challenges in assessing photosynthesis in non‐foliar organs.

### Photosynthetically active ear components

**Figure 3:**
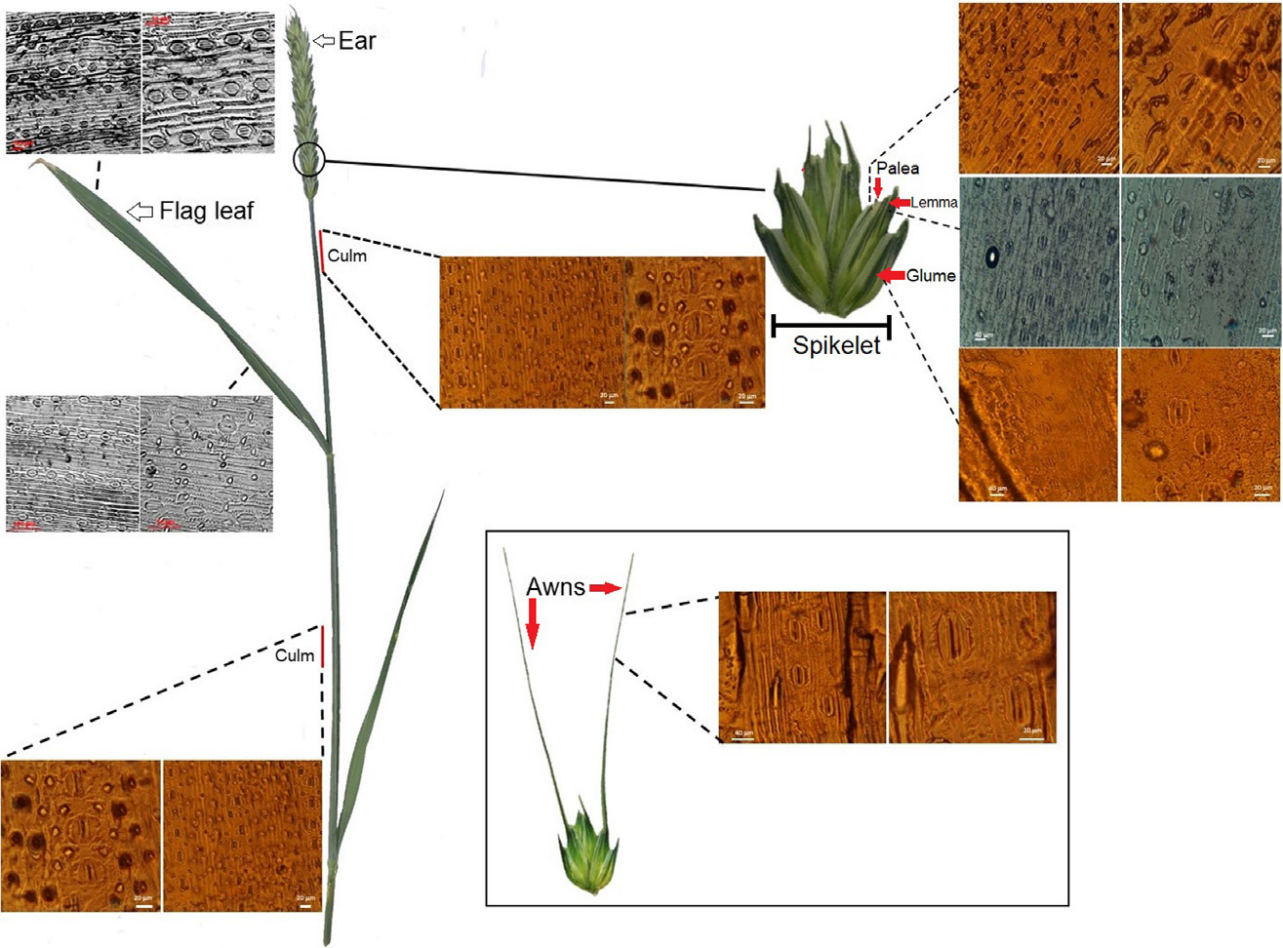
Schematic diagram and images of epidermal impressions illustrating stomatal anatomy and density in different components of wheat leaves (flag leaf), culm (stem) and ear (i.e. external surfaces of the glume, lemma and palea). The insert box provides an example of the stomatal density found on the awns of Soissons wheat.

The ear bracts (which consist of glume, lemma and palea) contain chlorophyll and possess stomata (Figure 3), and therefore have potential to fix atmospheric CO_2_ (Tambussi *et al.*, 2007). Genotypic variation in ear photosynthetic CO_2_ assimilation per unit area and contribution of ear photosynthesis to grain weight have been reported in the literature (Maydup *et al.*, 2014; Sanchez‐Bragado *et al.*, 2014). The exploitation of this variation might be of pivotal importance for cereal improvement. Several ear bracts have been considered putative locations of photosynthetic activity, with glumes, lemmas and awns considered the most photosynthetically active (Tambussi *et al.*, 2007; Hu *et al.*, 2019). In particular, the floral‐derived awns have been targeted as a trait to increase wheat yield owing to their high photosynthetic capacity of 7–35 µmol m^−2^ sec^−1^ (Hein *et al.*, 2016), and especially in view of the limited possibility to further increase assimilates partitioning to grains by manipulating the harvest index (Maydup *et al.*, 2014).

The seasonality of the post‐anthesis stages in cereals are often associated with increases in environmental stresses and severe water deficit conditions, leading to reduced yield. Numerous studies provide strong evidence that the ear possesses an elevated drought tolerance when compared with the flag leaf and highlight the ear as the main potential buffer for photoassimilate production under disadvantageous environments (Jia *et al.*, 2015). Additionally, the ear shows a lower transpiration rate than the flag leaf and a higher intrinsic WUE, confirmed by less negative δ^13^C values (Araus *et al.*, 1993; Tambussi *et al.*, 2007; Sanchez‐Bragado *et al.*, 2014; Vicente *et al.*, 2018). Xeromorphic characteristics in glumes, lemmas and awns of durum wheat have been observed, such as sclerenchymatous tissue and thick walls (Tambussi *et al.*, 2005). The same authors observed a higher osmotic adjustment and relative water content of the ear compared with the flag leaf under reduced water availability, leading to a sustained chlorophyll fluorescence signal. Similarly, ear bracts in barley maintained higher relative water content and gas exchange under water stress, compared with the leaf, as well as greater osmotic adjustment (Hein *et al.*, 2016).

Comparing awned and awnless lines under stress conditions showed higher ear intrinsic WUE (mainly driven by high photosynthetic activity for similar stomatal conductance (*g*_s_ per unit area) and photosynthetic capacity when awns were present, suggesting that awn photosynthesis also plays an important role when foliar tissue is reduced as a result of stress (Weyhrich, 1994; Weyhrich *et al.*, 1995). However, no differences in whole‐plant WUE and grain weight were found between these lines. Therefore, in this investigation, the higher photosynthetic capacity in the awns failed to contribute to yield. In contrast, a multi‐location field study on the effect of awns on wheat yield components showed that the presence of awns increased the grain size; however, this increase was compensated by a reduction in grain number (Rebetzke *et al.*, 2016), which was mainly attributed to the cost of awn setting. Assimilate partitioning to the floret is decreased in awned varieties through allocation to the rapidly growing awns, potentially followed by an associated reduction in floret fertility (Guo and Schnurbusch, 2016; Rebetzke *et al.*, 2016). It was concluded that awns are mainly useful under terminal drought conditions, owing to their elevated water stress tolerance that facilitates the maintenance of grain weight and a reduced number of shrivelled grains (screenings), compared with awnless lines, thus potentially providing higher economic yield and commercial value under such conditions. This was also confirmed by Maydup *et al. *(2014), who showed that awned varieties have higher ear photosynthesis, water status and ear water conductance compared with awnless varieties under water‐stress conditions in the field.

Genotypic variation of ear water‐stress tolerance has also been demonstrated by Li *et al. *(2017), where a stress‐tolerant wheat variety displayed a conservative water‐use strategy during post‐anthesis by reducing leaf transpiration while maintaining high levels of ear gas exchange. Vicente *et al. *(2018) postulated that water stress in wheat reduced the expression of photosynthetic genes (e.g. ATPase) in the flag leaf but not in the ear, and that the upregulation of respiration‐related genes, such as phosphoenolpyruvate carboxylase (PEPCase), 2‐oxoglutarate dehydrogenase complex (OGDC), alternative oxidase (AOX) and pyruvate kinase, was associated with the increased refixed CO_2_ in the ear organs. An observed upregulation of dehydrins (Abebe *et al.*, 2010), increased transcript levels of antioxidant enzyme genes (Vicente *et al.*, 2018), followed by high levels of antioxidant enzymes and low levels of ROS (Kong *et al.*, 2015) confirmed the higher drought tolerance of the ear and its importance as a main contributor to grain weight and, more broadly, grain yield under disadvantageous environmental conditions.

### Wheat endosperm and pericarp

Caley *et al. *(1990), followed by Tambussi *et al. *(2005), also proposed a possible role of the green pericarp in CO_2_ refixation. Although stomata are almost absent in the growing endosperm, suggesting limited gas‐exchange capacity, immunocytochemical analysis showed chloroplasts and Rubisco co‐localization in the green pericarp with elevated photosynthetic capacity (Kong *et al.*, 2016), which can account for up to 42% of the total photosynthetic activity of the ear (Evans and Rawson, 1970). Recent work reported that genes specific for the C4 pathways such as PEPC, NAD‐ME and NADP‐MDH are expressed in the cross and tube‐cell layer of the pericarp (Rangan *et al.*, 2016), agreeing with earlier studies that had already suggested the presence of C4 or C3–C4 intermediate metabolism in the ear (Ziegler‐Jöns, 1989; Imaizumi *et al.*, 1990; Li *et al.*, 2004; Jia *et al.*, 2015), potentially induced under water‐stress conditions. On the other hand, the following observations suggest limited evidence for a C4 pathway in the green pericarp and other ear organs: (i) oxygen sensitivity of CO_2_ assimilation rate of the ear (increased by up to 45% under conditions of 2% O_2_; Tambussi *et al.*, 2005; Tambussi *et al.*, 2007); (ii) high rates of CO_2_ assimilation through the CBC rather than conversion into C4 malate or aspartate (Bort *et al.*, 1995); and (iii) a lack of the specific C4 anatomy (Tambussi *et al.*, 2005), although future analyses are required to confirm this and it remains a topic of debate.

### The importance of stomata for ear photosynthesis

Several studies have demonstrated that the stomatal density in the flag leaf of wheat varies between 40 and 90 mm^−2^ (e.g. Faralli *et al.*, 2019a), and that in ear organs the stomatal density can be either higher (Kong *et al.*, 2015) or drastically lower (Tambussi *et al.*, 2005) than in the leaf. Furthermore, different stomatal densities and distributions have been reported on both the ventral and the dorsal sides of the glume and the lemma (Figure 3), with the lemma showing variable density depending on the shading area of the neighbouring glume (Tambussi *et al.*, 2005). As the growing endosperm releases respired CO_2_, the presence of stomata in the internal surface of glumes and lemmas is evidence of CO_2_ recycling capacity. As reported for fruit (see above), several studies have demonstrated large rates of refixation of respiratory CO_2_ in the ear (Bort *et al.*, 1996), which can contribute up to 79% of the sucrose accumulated in bracts (Gebbing and Schnyder, 2001). The refixation capacity has several advantages, in particular: (i) respiratory CO_2_ losses are minimized; and (ii) photosynthetic metabolism is fully independent of the environment.

**Figure 4:**
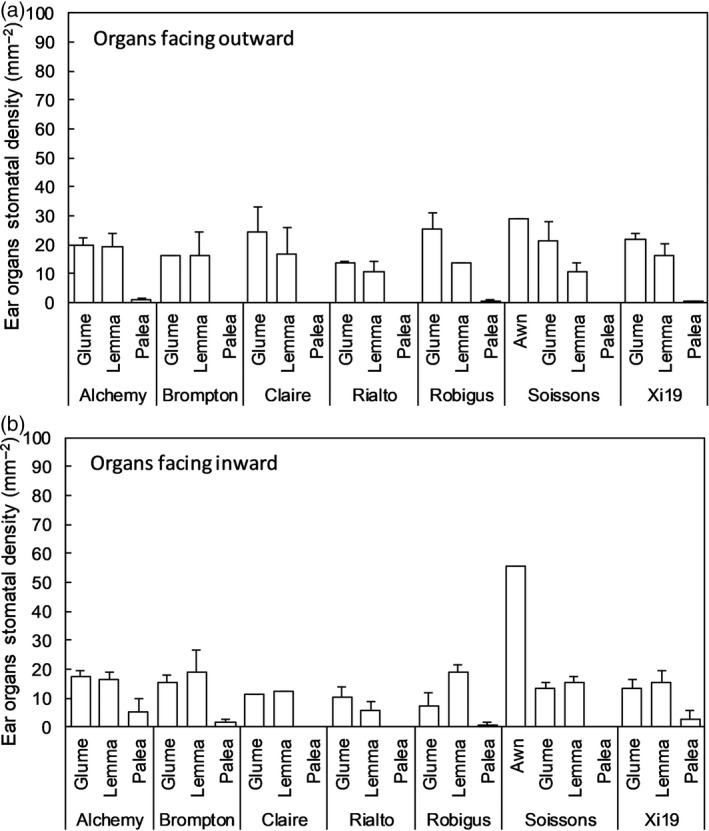
Stomatal density of ear organs (glume, lemma, palea and awns, when present) for seven bread wheat elite cultivars collected after anthesis (a and b). Wheat plants were grown in a glasshouse and ears were harvested at the end of anthesis (i.e. GS69). Stomatal analysis was carried out as described by Faralli *et al.* 2019b. Data are means ± standard errors of the mean (*n* = 2–7).

**Figure 5:**
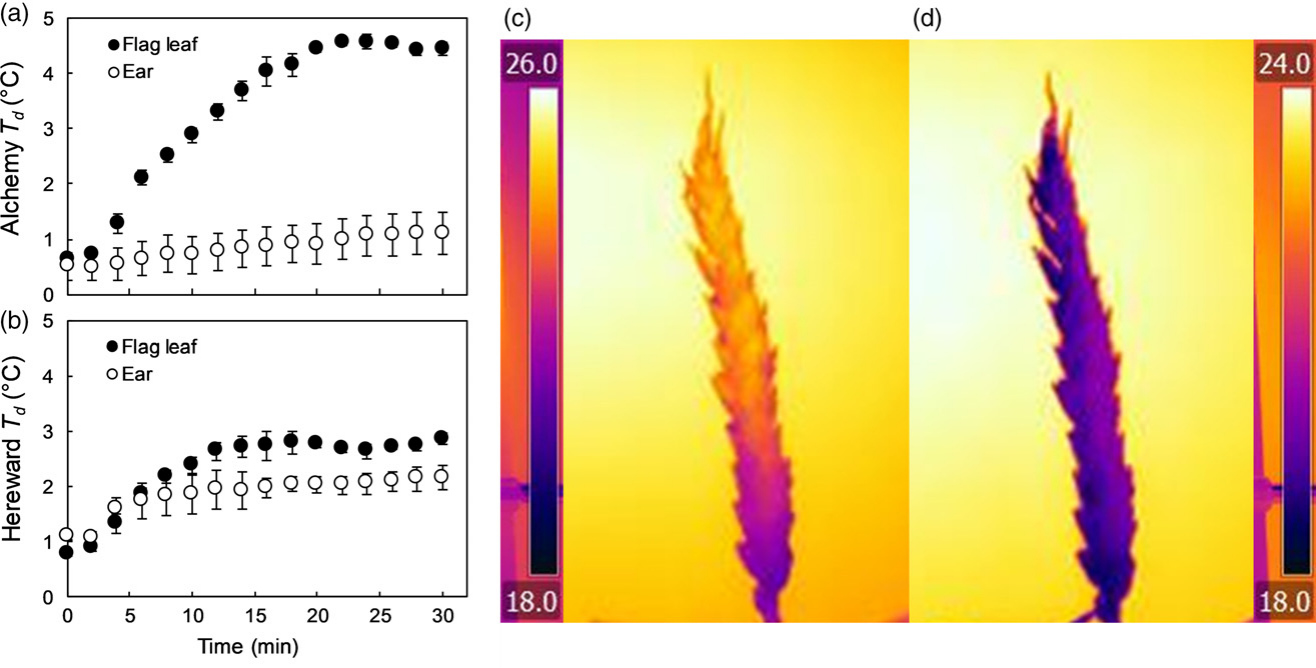
Temperature differential between the dry reference and either the flag leaf or the ear of two bread wheat varieties grown in glasshouse conditions (*n* = 4 cv. Alchemy and Hereward) subjected to a step change in light (from 100 to 1000 µmol m^−2^ s^−1^) and maintained under high light conditions for 30 min. Thermal images of a wheat ears (c, d) following the step increase in light intensity shows significant temperature differences in plants subjected to 10 min (c) or 25 min (d) of illumination, illustrating stomatal functioning in increased evaporative cooling.

Genotypic variation in stomatal distribution in glumes and lemmas, and on the different sides, also exists in current elite bread wheat cultivars (Figures 3 and 4), which suggests different strategies for atmospheric CO_2_ assimilation or CO_2_ refixation that could be further exploited for ear gas‐exchange optimization. In general, high stomatal densities are reported on the external side of glumes (up to 32 mm^−2^) and awns (up to 70 mm^−2^), with lower numbers found in lemmas (between 20 and 10 mm^−2^) and absent in paleas (Figure 4). The stomatal density on the internal surfaces are comparable for glumes and lemmas (between 20 and 9 mm^−2^), but are almost absent in paleas. It has been reported that stomatal functionality may be strongly limited in the ear by: (i) the mechanical constraint induced by the growing grains inside the florets; and (ii) by the accumulation of waxes preventing guard cells opening and closing (Araus *et al.*, 1993) and hence limiting photosynthetic CO_2_ uptake, especially during the late grain filling stage. Figure 5 shows thermal images from the ear and flag leaf of two wheat varieties, and reveals that although the temperature regulation of the ear is significantly lower than that of the flag leaf (i.e. lower transpiration rate), the ear stomata are responsive and open when subjected to a transition from low to high light conditions. In the ear the two cultivars also differ in the magnitude and rapidity of stomatal opening (Faralli *et al.*, 2019b), suggesting potential genotypic variation, driven by either differences in wax accumulation (Araus *et al.*, 1993) or variation in stomatal size, density and distribution, as well as functional differences. Indeed, in glasshouse experiments, six recombinant inbred lines grown under conditions of heat and water stress showed the presence of cooling capacity in the ear at early anthesis (i.e. before pollen release) (Steinmeyer *et al.*, 2013). With the elevated sensitivity of pollen to high temperatures, ear stomatal dynamics and the overall evaporative cooling capacity may be important novel traits for increasing stress tolerance by protecting pollen viability and minimizing floret damage at anthesis. Indeed, at the reproductive stage, stress tolerance in crops is based on both the ability to produce viable pollen and to ‘shield’ the pollen from environmental stresses (i.e. reducing the temperature of reproductive organs with a high transpiration rate) (Steinmeyer *et al.*, 2013). In addition, enhancing stomatal regulation and transpiration may increase assimilate translocation to the developing grains and the remobilization of resources, and could be considered as an additional target for increasing yield potential.

## Challenges associated with measuring photosynthesis in non‐foliar tissue

**Figure 6:**
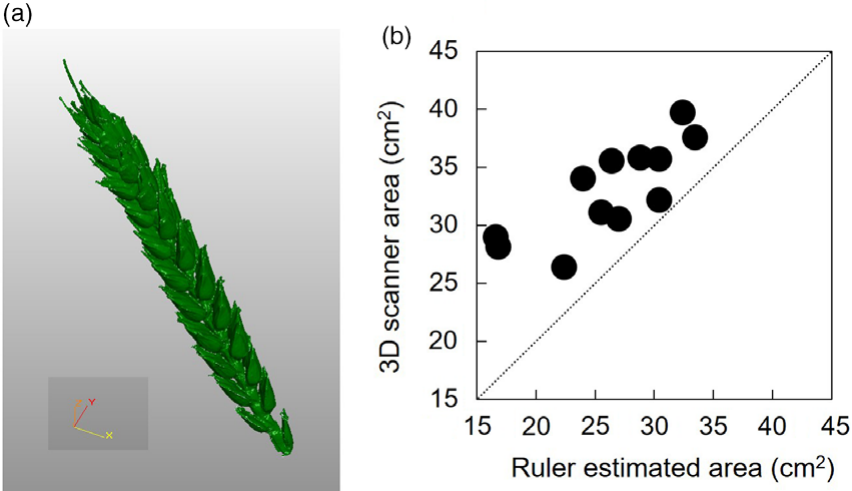
(a) Example of a detailed assessment of wheat ear area and volume using a 3D scanner approach. (b) Example of the underestimation of ear area using a ruler‐based approach compared with a detailed 3D scanner estimation. Wheat plants (cv. Cadenza) were grown in a glasshouse and primary and secondary ears were harvested at different times and over three periods after anthesis. The area was estimated with a ruler by measuring ear length and width of all the four surfaces and then the same ear was assessed with a 3D scanner (*n* = 4 for each harvest).

Further experimental evidence is needed to fully understand the mechanisms involved in photosynthetic activity of the ear and other non‐foliar photosynthetic organs. There are challenges associated with measuring photosynthesis in non‐laminar tissues using the standard approaches used for leaves. For example, most leaf‐level measurements of CO_2_ uptake are conducted using infrared gas analysis (IRGA), which requires the material to be enclosed in a sealed chamber, with the differences in gas fluxes in and out of the chamber being assessed. Using such approaches for non‐leaf material represents challenges, including: (i) the small size of commercially available leaf gas‐exchange chambers; (ii) the complication of refixation of respiratory CO_2_ in determining gas differentials; and (iii) the complexity of ear architecture in wheat, making the normalization of gas‐exchange data per unit area particularly difficult and leading to strong uncertainty in the absolute values. New methodologies are needed and should be implemented to assess ear gas exchange and organ contribution to grain weight. For instance, 3D scanners help to refine the estimation of area, in particular in view of the consistent underestimation (and thus gas‐exchange overestimation) that occurs with standard techniques (e.g. using a ruler; Figure 6). Additionally, the design and development of bespoke chambers is required to enclose an entire ear or fruit to allow the assessment of whole‐organ gas exchange. Such chambers present further challenges that arise from the large volumes required, which can lead to slow gas mixing and difficult temperature control. In addition, although saturated light can be provided in large cuvettes for all surfaces, the shading effects from neighbouring organs, e.g. spikelet morphology and distance between spikelets, may lead to additional sources of error. Chlorophyll fluorescence has been shown to be a good candidate for ear photosynthetic assessment (Tambussi *et al.*, 2005; Maydup *et al.*, 2012), and combined with gas exchange (McAusland *et al.*, 2013) may help to dissect the proportion of photosynthesis relying on the refixation of respiratory CO_2_ from atmospheric CO_2_, as well as determining differences in the O_2_ sensitivity of various genotypes.

Defoliation, inhibition of photosynthesis through shading and herbicide application are some of the most commonly used approaches to evaluate the contribution of ear photosynthesis to yield (Sanchez‐Bragado *et al.*, 2016). Although these approaches may be useful to evaluate genotypic variation, they are likely to induce compensatory mechanisms (and potentially overestimations). Sanchez‐Bragado *et al. *(2016) suggested carbon isotope discrimination as an alternative for assessing ear photosynthetic traits. In addition, owing to the Rubisco discrimination of ^13^C and because of the lack of carbon discrimination in PEPC, the isotopic signature may help to discern potential variation between the C3 and C4 pathways (Hu *et al.*, 2019). It must be recognized that almost all the approaches outlined above lack the advantage of high throughput and are generally considered time consuming and laborious, and this therefore limits their use for screening large populations or samples for ear photosynthetic phenotypes. There is no doubt that improvement in experimental procedures along with further advances in high‐throughput approaches for screening ear photosynthesis will increase our understanding of ear photosynthetic activity and therefore help to design new cereal varieties with elevated yield potential and stability.

## Conclusion

**Figure 7:**
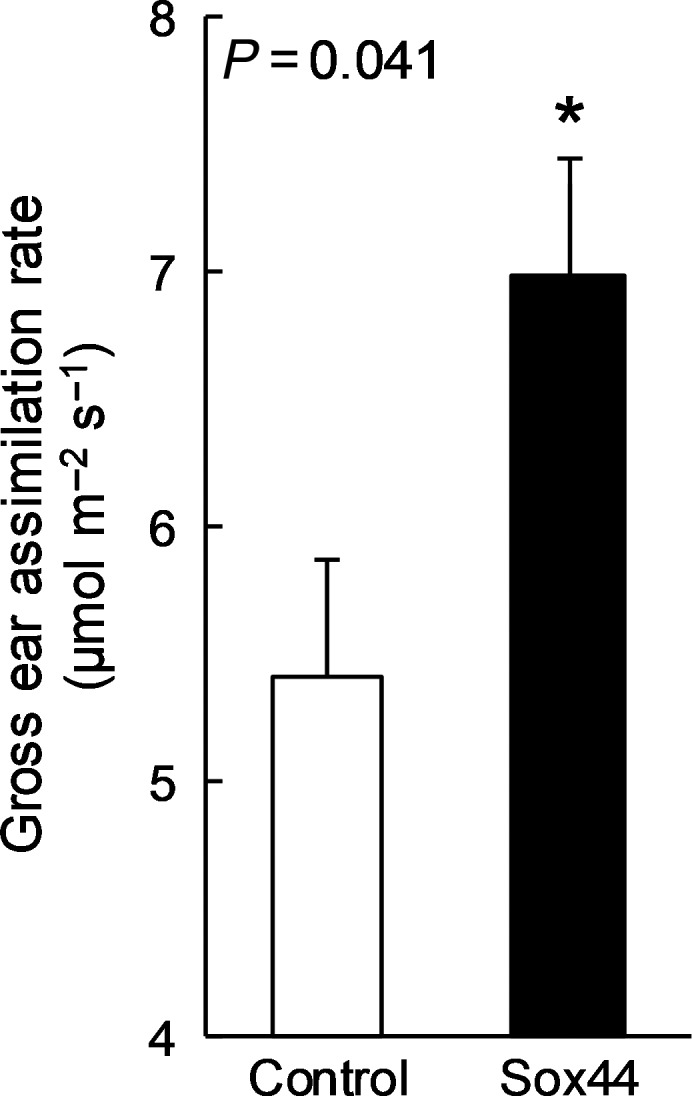
Gross assimilation rate calculated as the sum of light‐saturated assimilation rate and dark respiration of wheat ears (*n* = 5) of control cv. Cadenza plants and transgenic plants overexpressing SBPase (Driever *et al.*, 2017). Data were collected post‐anthesis in glasshouse‐grown plants with a Licor 6400XT mounted with a bespoke cuvette ensuring saturating light (1000 μmol m^−2^ sec^−1^) and a 25°C block temperature.

Although most studies examining photosynthesis have focused on leaf‐level measurements, including current approaches to improve photosynthesis, the contribution that other green tissues make to total photoassimilates has largely been ignored. As highlighted above, these green tissues contribute significantly to plant development, growth and yield, and therefore present novel opportunities for exploitation to improve productivity. The fact that the full spectrum of light harvest, electron transport and CBC proteins and transcripts are found in non‐foliar tissues (Barsan *et al.*, 2010; Barsan *et al.*, 2012; Sui *et al.*, 2017; Vicente *et al.*, 2018) offers the potential to manipulate non‐foliar photosynthetic pathways to increase rates of photosynthesis using similar approaches to those currently being employed in leaves (for a review, see Simkin, 2019 and Simkin *et al.*, 2019). For example, recent experiments in transgenic wheat with increased activity of the CBC enzyme SBPase, driven by a constitutive promotor (Driever *et al.*, 2017), revealed increased gross photosynthesis in the ears of mutant plants relative to the wild‐type control (Figure 7). It is therefore possible that the overall increase in yield of plants overexpressing SBpase reported by Driever *et al. *(2017) may have been achieved in part by an increase in ear‐derived assimilates, although this would require further investigation. Such studies highlight the potential benefits of improving photosynthesis in organs other than leaves for improving crop productivity and yield. Furthermore, as photosynthesis provides the building blocks for many downstream products and metabolites, modifying photosynthetic processes in fruits, for example, offers the potential to alter fruit quality and nutritional value.

A major difference between leaf and non‐leaf tissues is the primary source of CO_2_ for CBC (atmospheric versus respiratory), and therefore the manipulation of stomatal density or function presents an additional avenue to manipulate photosynthetic processes in some tissues, e.g. wheat ears. For example, increasing stomatal density or aperture could result in increasing assimilation by removing diffusional constraints and increasing the flux of atmospheric CO_2_ to the site of carboxylation; however, such an approach would also facilitate the leakage of respiratory CO_2_ (Sui *et al.*, 2017), which has been demonstrated to be of greater importance in some organs. Alternatively, increased stomatal density in wheat ears could improve evaporative cooling, thereby maintaining assimilation rates under elevated temperatures, assuming a similar temperature sensitivity of photosynthesis in wheat ears and leaves (Scafaro *et al.*, 2012; Scafaro *et al.*, 2016; Perdomo *et al.*, 2017). On the other hand, this ‘risky’ behaviour might increase the possibility of early ear dehydration under severe terminal stress conditions, although further experimental evidence is required to support this theory. Stomatal behaviour and transpiration in ears may also provide a key role in the translocation of photoassimilates to the ear, and therefore altering *g_s_* could assist with sink–source relationships. Although stomatal behaviour is important for photosynthesis, it should be acknowledged that stomatal pores are also an important component of non‐leaf tissues to facilitate drying, which is essential for the dispersal of spores and seeds (e.g. stomata in the spore capsules of moss; Merced and Renzaglia, 2013; Chater *et al.*, 2016). Before such novel targets for improved photosynthesis can be exploited, a better understanding of the contribution of non‐foliar photosynthesis to yield and quality (particularly under conditions of stress) and the role of stomata in these processes is needed.

## Conflict of interest

There are no conflicts of interest to declare.

## Author contributions

AJS, MF and TL all wrote the article and contributed to the figures. Data presented on the SBPase wheat are from work carried out by MF, AJS, TL and Christine Raines.

## Data Availability

Data presented within this review are example data sets that are not publicly available; please contact T.L. to request access to any data.
